# High tandem repeat content in the genome of the short-lived annual fish *Nothobranchius furzeri*: a new vertebrate model for aging research

**DOI:** 10.1186/gb-2009-10-2-r16

**Published:** 2009-02-11

**Authors:** Kathrin Reichwald, Chris Lauber, Indrajit Nanda, Jeanette Kirschner, Nils Hartmann, Susanne Schories, Ulrike Gausmann, Stefan Taudien, Markus B Schilhabel, Karol Szafranski, Gernot Glöckner, Michael Schmid, Alessandro Cellerino, Manfred Schartl, Christoph Englert, Matthias Platzer

**Affiliations:** 1 Leibniz Institute for Age Research - Fritz Lipmann Institute, Beutenbergstr., 07745 Jena, Germany; 2 Department of Physiological Chemistry I, University of Würzburg, Biozentrum, Am Hubland, 97074 Würzburg, Germany; 3 Department of Human Genetics, University of Würzburg, Biozentrum, Am Hubland, 97074 Würzburg, Germany; 4 Current address: Department of Medical Microbiology, Leiden University Medical Centre, 2300 RC Leiden, The Netherlands; 5 Current address: Institute of Clinical Molecular Biology, University Hospital Schleswig-Holstein, Campus Kiel, Schittenhelmstr., 24105 Kiel, Germany

## Abstract

**Background:**

The annual fish *Nothobranchius furzeri *is the vertebrate with the shortest known life span in captivity. Fish of the GRZ strain live only three to four months under optimal laboratory conditions, show explosive growth, early sexual maturation and age-dependent physiological and behavioral decline, and express aging related biomarkers. Treatment with resveratrol and low temperature significantly extends the maximum life span. These features make *N. furzeri *a promising new vertebrate model for age research.

**Results:**

To contribute to establishing *N. furzeri *as a new model organism, we provide a first insight into its genome and a comparison to medaka, stickleback, tetraodon and zebrafish. The *N. furzeri *genome contains 19 chromosomes (2n = 38). Its genome of between 1.6 and 1.9 Gb is the largest among the analyzed fish species and has, at 45%, the highest repeat content. Remarkably, tandem repeats comprise 21%, which is 4-12 times more than in the other four fish species. In addition, G+C-rich tandem repeats preferentially localize to centromeric regions. Phylogenetic analysis based on coding sequences identifies medaka as the closest relative. Genotyping of an initial set of 27 markers and multi-locus fingerprinting of one microsatellite provides the first molecular evidence that the GRZ strain is highly inbred.

**Conclusions:**

Our work presents a first basis for systematic genomic and genetic analyses aimed at understanding the mechanisms of life span determination in *N. furzeri*.

## Abstract 2

A genomic analysis of the annual fish Nothobranchius furzeri, a vertebrate with the shortest known life span in captivity and which may provide a new model organism for aging research.

## Background

Studies in invertebrate model organisms such as yeast and worm have identified a number of genes and pathways that regulate life span and aging [1], and some of these are conserved across taxa [2-5]. While this work has been crucial to elucidate common aging related pathways like insulin/insulin-like growth factor signaling [6-8], in many cases experimentally proven relevance for invertebrate genes/gene products cannot be reproduced in vertebrates. Also, the comparatively long life span of vertebrate model organisms poses a difficulty for testing of findings initially obtained in invertebrates - for example, a life expectancy of several years like that of mouse, rat, or zebrafish renders experimental analyses of potentially life extending drugs difficult.

The Turquoise killifish, *Nothobranchius furzeri*, might represent an alternative vertebrate model for the study of aging [9]. The fish inhabit seasonal ponds in South-East Africa and were first captured in 1968 in the Game Reserve Gona Re Zhou (GRZ) in Zimbabwe [10]. In 1975, only two pairs of the direct descendants of these fish were left. One of these pairs was then used for breeding, mainly to preserve the species for the killifish community [11]. Offspring of these fish have since been maintained by dedicated hobbyists and in the following are referred to as the GRZ strain. The maximum life span of GRZ fish was previously found to be 12-13 weeks in captivity [12-14]. In our facility, GRZ fish exhibit a maximum life span of 16 weeks [15]. The fish show explosive growth and early sexual maturation, and with advancing age typical aging related features such as a decline in learning/behavioral capabilities as well as expression of aging biomarkers are observed [16,17]. Furthermore, GRZ fish are susceptible to life span modulation. Maximum life span is significantly prolonged by moderately decreased water temperature and treatment with resveratrol, both of which are characterized by delayed onset of cognitive decline and expression of aging biomarkers [13,14]. In contrast to the GRZ strain established from the game reserve in Zimbabwe, recently established isolates of *N. furzeri *populations from southern Mozambique differ in life span and time of expression of age-related traits, which presumably reflects adaptation to the seasonal duration of their respective ponds [18]. The maximum life span of these recent isolates is 25-32 weeks. Although this is twice as long as found for the GRZ strain, it is still exceptionally short compared to other vertebrates. It also does not seem to change considerably in captivity - for example, maximum life span is 32 weeks in the first captive generation of a southern *N. furzeri *population collected in 2004 and remains at 31 weeks in the sixth captive generation of the derived strain [15], which is currently bred in our facility and was termed *N. furzeri *MZM-0403 [18]. In addition, there are several other African *Nothobranchius *species, which live longer than *N. furzeri*, including *N. kunthae *(37 weeks) and *N. guentheri *(52 weeks, reviewed in [9]), opening up the possibility to study naturally occurring aging phenotypes both in *N. furzeri *and across *Nothobranchius *species.

To date very little is known about the genetic makeup of *N. furzeri *and at present one cannot exclude that private mutations or inbreeding cause the exceptionally short life span of the GRZ strain. However, the presence of aging related biomarkers and changes in their expression in response to external stimuli [14,16], as well as a similarly short life span and visible symptoms of old age reported for GRZ fish 33 years ago [11], argue in favor of the action of common mechanisms of life span determination in this strain.

In order to support establishing *N. furzeri *as a model organism for age research, we provide here a first insight into its cytogenetic and genomic characteristics, including karyotype, genome size/composition, phylogenetic positioning and genetic variability. We compare its genomic features to those of medaka (*Oryzias latipes*), stickleback (*Gasterosteus aculeatus*), tetraodon (*Tetraodon nigroviridis*) and zebrafish (*Danio rerio*). These species serve as models in many areas of contemporary research, such as genome evolution (tetraodon), developmental biology and genetics (medaka, zebrafish), and speciation (stickleback). Genome projects are completed or well underway; whole genome analyses have been published for tetraodon and medaka [19,20], and preliminary genome assemblies have been provided for stickleback and zebrafish [21]. Clearly, the sequences facilitate large scale and systematic studies [22-24].

Our work is a first step towards a systematic identification of genes and biochemical pathways involved in life span determination in *N. furzeri*. Combined with the genetic resources for the aforementioned fish species it also forms a basis to make full use of *N. furzeri *as a model organism for the study of aging.

## Results and discussion

### Cytogenetic characteristics

**Figure 1:**
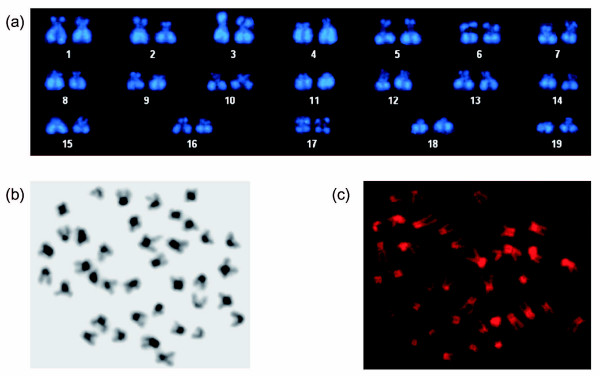
Cytogenetic features of *N. furzeri*. **(a) **Karyotype of DAPI-stained chromosomes of a female *N. furzeri *of the GRZ strain. Note the absence of bright staining at the centromeric regions. Four pairs of chromosomes (1, 6, 9, 17) are metacentric, three pairs (16, 18, 19) are acrocentric and the remaining 12 pairs are subtelo-/submetacentric. **(b) **C-banded karyotype of a female *N. furzeri *GRZ reveals centromeric heterochromatin in most chromosomes. **(c) **Mithramycin staining results in bright fluorescence of centromeres, which is due to G+C enriched heterochromatin.

**Figure 2:**
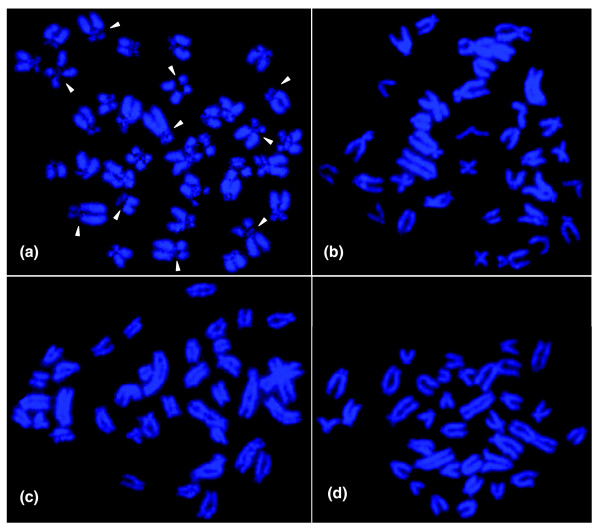
Chromosomes of four African *Nothobranchius *species. DAPI stained chromosomes of **(a) **a female specimen of the *N. furzeri *GRZ strain, **(b) **a male specimen of the sympatric species *N. orthonotus*, and **(c) **a male specimen of the allopatric species *N. hengstleri *and **(d) ***N. eggersi*, respectively. Note the dull DAPI fluorescence at centromeric regions in *N. furzeri *chromosomes (indicated by arrowheads), which is indicative of the presence of G+C-rich constitutive heterochromatin and not observable in the three closely related *Nothobranchius *species.

The chromosome number of *N. furzeri *is 2n = 38 and includes four pairs of metacentric, three pairs of acrocentric and twelve pairs of subtelo-/submetacentric chromosomes (Figure 1a). Based on morphology, there do not seem to be clearly differentiated sex chromosomes. Specific heterochromatin staining indicates that the cytological organization of the *N. furzeri *genome is highly structured as is evident from the presence of large blocks of C-banding positive heterochromatin in the centromeric region of most chromosomes (Figure 1b). Accumulation of heterochromatin in other chromosomal sites cannot be detected. To evaluate the composition of heterochromatin, we performed base specific fluorochrome staining on metaphase chromosomes. Staining with the A+T specific dye DAPI resulted in poor fluorescence of centromeric regions (Figures 1a and 2a). Conversely, staining with mithramycin, which shows high affinity for G+C-rich DNA, generated bright fluorescence in most centromeric regions that are DAPI dull (Figure 1c). This indicates that constitutive heterochromatin in *N. furzeri *is G+C-rich. The analysis of closely related African *Nothobranchii*, including the sympatric species *N. orthonotus *as well as two allopatric species from Tanzania, *N. hengstleri *and *N. eggersi*, does not indicate a comparably structured genome organization (Figure 2b–d). Thus, at the cytological level, a compartmentalization of the *N. furzeri *genome is apparent which is likely caused by substantial differences in DNA composition. This seems unusual since fish genomes are generally characterized by a very limited compositional heterogeneity [25,26].

### Genome size

We sequenced 5.4 Mb of the short-lived strain *N. furzeri *GRZ, the long-lived, recently wild-derived strain *N. furzeri *MZM-0403, and the long-lived closely related species *N. kunthae *using a whole genome shotgun approach and Sanger sequencing (Table 1). To assess the genome size of *N. furzeri *based on the sequences, we assumed that the number of its protein coding genes does not differ significantly from that of other fish species, as was previously suggested for vertebrates [27]. We identified sequences containing protein coding information in the 5.4 Mb of both *N. furzeri *strains by BLASTX searches in Swiss-Prot/TrEMBL, and did the same for medaka, stickleback, tetraodon and zebrafish, for which we extracted adequate genomic samples from public databases. Based on the reported genome sizes of the latter four fish species, we then deduced the genome size of *N. furzeri*. In detail, 444 (8%) of the 5,540 *N. furzeri *GRZ sequences and 443 (8%) of the 5,686 MZM-0403 sequences show significant similarity (*p *< 10^-10^) to protein coding genes. Respective sequences comprise 31%, 20%, 13% and 11% of the tetraodon, stickleback, medaka and zebrafish genomic samples, respectively, which corresponds to a genome size of 1.59-1.92 Gb for *N. furzeri *(Additional data files 1 and 2). For experimental confirmation, we performed flow cytometry measurements using DAPI and propidium iodide (PI). DAPI, which preferentially stains A+T rich DNA segments, yields a DNA content of 2.33 pg/diploid cell while the non-base-specific PI staining results in 3.11 pg/diploid cell (Additional data file 3). In light of the large blocks of G+C-rich heterochromatin observed in our cytogenetic studies, the value obtained with PI is likely the correct one since it is based on a dye that does not depend on DNA composition, whereas DAPI staining most probably results in an underestimation of DNA content [28]. The value obtained with PI corresponds well with our sequence based estimate; that is, it is equal to a genome size of approximately 1.5 Gb. The 5.4 Mb thus represents roughly 0.3-0.5% of the *N. furzeri *genome.

**Table 1:** Whole genome shotgun sequences

	*N. furzeri*			*N. kunthae*
		
	GRZ: Sanger sequencing*	GRZ: pyrosequencing^†^	MZM-0403: Sanger sequencing*	Sanger sequencing*
Number of sequence contigs	5,540	1,095,308	5,686	6,273
Average length ± SD (bp)	968 ± 446	102 ± 15	948 ± 408	855 ± 432
Range of length (bp)	101-2,685	36-230	100-2,699	100-2,738
				
Total sequence (bp)	5,364,828	111,563,506	5,364,828	5,366,245
Number of uncalled bases	6,661	32,643	3,809	3,786
Percentage of uncalled bases	0.12	0.03	0.07	0.07
				
Percentage of bases with Phred^‡ ^≥40	84.1	1.3	75.1	76.1
Percentage of bases with Phred ≥30	89.5	22.5	83.6	85.0
Percentage of bases with Phred ≥20	93.2	89.2	89.7	91.2
				
G+C content (%)	44.9	44.3	44.3	44.9

*Sanger sequencing was performed on ABI 3730xl machines. ^†^Pyrosequencing was performed on Roche/454 GS20 sequencers; sequences were not assembled. ^‡^Phred ≥40 corresponds to at least 99.99% accuracy; Phred ≥30 to 99.9% accuracy; Phred ≥20 to 99% accuracy. SD, standard deviation.

Based on these data, the *N. furzeri *genome is likely at least half the size of the human genome, bigger than the four other fish genomes, and has less chromosomes. At 1.4 Gb (25 chromosomes) [29], the zebrafish genome is slightly smaller, while medaka (1 Gb, 24 chromosomes [20]), stickleback (0.7 Gb, 21 chromosomes [30]) and tetraodon (0.4 Gb, 21 chromosomes [31]) genomes are considerably more compact.

### Genome composition

**Figure 3:**
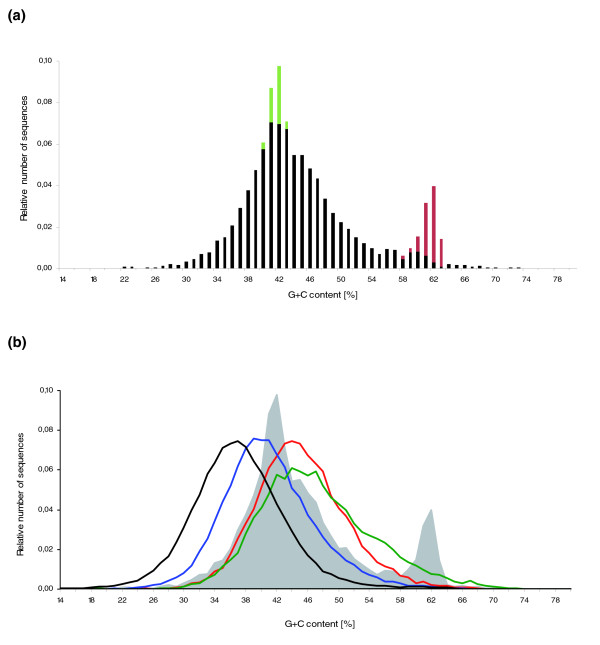
G+C content distribution of *N. furzeri *compared with medaka, stickleback, tetraodon and zebrafish. **(a) **Histogram of the G+C content of the 5.4 Mb genomic sample of *N. furzeri *GRZ. The average G+C content is 44.9%. Note G+C distortions, which are seen in a second peak at approximately 62% G+C and an unusually high number of sequences with approximately 41% G+C. Green: sequences containing the most frequent G+C poor 348-nucleotide satellite repeat. Red: sequences containing the most frequent G+C-rich 77-nucleotide minisatellite repeat. **(b) **G+C content distribution of ten samples of random sequence sets of zebrafish (black), medaka (blue), stickleback (red) and tetraodon (green), respectively. Each data set of the four fish genomes is shown with respect to sequence length distribution and occupied genomic fraction similar to the *N. furzeri *GRZ 5.4 Mb sample, which, for comparison, is shown as a grey area. Average G+C content values are 36.6% for zebrafish, 40.5% for medaka, 44.6% for stickleback and 46.6% for tetraodon.

The G+C content of the 5.4 Mb sample of *N. furzeri *GRZ is 44.9%. Interestingly, approximately 10% of the sequences have a G+C content of 60% or higher; in Figure 3a this is indicated by a second peak at approximately 62% in a plot of number of sequences against G+C content. The same unusual G+C content distribution is seen in the recently wild-derived *N. furzeri *strain MZM-0403 (Additional data file 4). To test whether this represents an artifact introduced by preferential propagation of G+C-rich sequences in *Escherichia coli *[32] during library preparation, we performed whole genome shotgun sequencing using Roche/454 Life Sciences technology, which does not involve cloning and amplification in bacterial systems [33], for *N. furzeri *GRZ. In this second GRZ genomic sequence sample, the G+C content is 44.3% (111.6 Mb; Table 1). Similar to the Sanger sequences, approximately 10% of the 454 sequences show a G+C content of at least 60%, which indicates that there is not a strong cloning bias in the genomic libraries we initially sequenced. Taken together, our sequence data suggest that a distinct G+C-rich fraction is present in the *N. furzeri *genome. A similar fraction does not seem to exist in medaka, stickleback, tetraodon and zebrafish (Figure 3b) and is absent as well in the closely related species *N. kunthae *(Additional data file 4).

To assess the extent to which our sample-based estimation reflects the G+C content of the *N. furzeri *genome, we analyzed entire genomes as well as adequate genomic samples of medaka, stickleback, tetraodon and zebrafish. We found that the G+C content estimates of genomes and genomic samples are essentially the same (Table 2). Our calculations are in agreement with previously published data; we estimated a G+C content of 46.4% for tetraodon and reported values are 45.5% [31] and 46.4% [20]; similarly, 40.3% was reported for medaka [20] and we found it to be 40.5%. Thus, the G+C content of *N. furzeri *is likely 44-45%, which is similar to stickleback (44.6%), slightly lower than in tetraodon (46.4%) and considerably higher than in medaka (40.5%) and zebrafish (36.6%). Based on genomic sequences, an inverse correlation of genome size and G+C content was recently found for the latter four fish species [34], which confirmed previous experimental results showing that small genomes are generally associated with high G+C content and *vice versa *[25]. *N. furzeri *would seem an exception as its G+C content is nearly as high as that of tetraodon, which has a four times smaller genome. The sequence fraction in the *N. furzeri *genome with the high G+C content (≥60%) increases the global G+C content rather slightly - the value is 43.1% if these sequences are excluded - and it seems that these sequences occupy defined chromosomal regions (see below).

**Table 2:** G+C and repeat content of *N. furzeri*, *N. kunthae*, tetraodon, stickleback, medaka, and zebrafish

	N. furzeri*		*N. kunthae*^†^	Tetraodon^‡^	Stickle back^‡^	Medaka^‡^	Zebrafish^‡^
						
	GRZ	MZM- 0403					
G+C content of samples (%)	44.9	44.3	44.9	46.6 ± 0.2	44.6 ± 0.1	40.5 ± 0.1	36.6 ± 0.0
Genome-wide^§ ^(%)	NA	NA	NA	46.4	44.6	40.5	36.6
							
Repeat content of samples (%)	45.3	45.1	45.1	06.9 ± 0.4	6.6 ± 0.3	15.3 ± 0.6	40.4 ± 0.4
Genome-wide^¶ ^(%)	NA	NA	NA	5.4	NA	17.5	NA
							
Tandem repeats (%)	20.6	20.6	10.6	03.6 ± 0.3	2.1 ± 0.2	1.7 ± 0.2	5.0 ± 0.2
Microsatellites (%)	0.9	0.8	1.1	01.1 ± 0.1	0.8 ± 0.1	0.2 ± 0.0	2.0 ± 0.1
Most abundant^¥^, unit size (bp)	77	77	31	10	317	20	32
Content (%)	9.5	8.3	1.2	0.7	0.1	0.1	0.1
							
Interspersed repeats (%)	24.7	24.5	34.5	03.4 ± 0.3	4.5 ± 0.4	13.6 ± 0.6	35.4 ± 0.4
Known repeats (%)	8.9	6.9	09.0	3.1 ± 0.2	3.6 ± 0.2	7.0 ± 0.4	30.6 ± 0.2
Non-LTR retrotransposons	5.2	5.1	07.3	1.2 ± 0.2	1.4 ± 0.3	2.8 ± 0.2	5.8 ± 0.2
LTR retrotransposons	1.4	0.8	01.0	0.2 ± 0.1	0.6 ± 0.1	0.6 ± 0.1	2.3 ± 0.2
DNA transposons	1.7	1.3	01.4	0.6 ± 0.1	0.7 ± 0.1	3.2 ± 0.3	20.9 ± 0.3
Unclassified repeats (%)	15.8	17.6	25.5	0.3 ± 0.1	0.9 ± 0.2	6.6 ± 0.4	4.8 ± 0.3

*The 5.4 Mb genomic sample of strains GRZ and MZM-0403 generated by Sanger sequencing representing approximately 0.3-0.5% of the *N. furzeri *genome. ^†^The 5.4 Mb genomic sample of closely related species *N. kunthae*. ^‡^Mean and standard deviation of ten samples of random genomic sequence sets with each set representing 0.4% of the respective genome. ^§^Calculations based on respective genome reference assemblies at *Ensembl *[21]. ^¶^According to Roest Crollius *et al. *for tetraodon [31], and Kasahara *et al. *for medaka [20]. ^¥^Microsatellites excluded; value for concatenation of ten random sequence sets of tetraodon, stickleback, medaka, and zebrafish, respectively. LTR, long terminal repeat; NA, not available.

While to our knowledge there is no evidence for a direct influence of nuclear genome composition on the life span of a vertebrate species, some reports correlate life span with the composition of proteins encoded by the mitochondrial genome. For example, Rottenberg [35] found that rates of amino acid substitution per site in mitochondrial DNA of mammals are positively correlated with longevity of a genus and suggested that the evolution of longevity drove the accelerated evolution of peptides encoded by mitochondrial DNA. Moosmann and Behl [36] showed that the frequency with which cysteine is encoded by mitochondrial DNA is a specific indicator for longevity; that is, longevity is associated with a depletion of mitochondrial cysteine in aerobic species. It will be very interesting to analyze the cysteine content in mitochondrially encoded proteins in comparison to nuclear proteins in *N. furzeri *strains/*Nothobranchius *species with different life spans.

### Repeats

Repetitive elements can be grouped into the main classes 'tandem repeats', 'transposon-derived interspersed repeats', 'processed pseudogenes' and 'segmental duplications'. Because our genomic samples comprise rather small, fragmented fractions of the *N. furzeri *genome, it is impossible to identify pseudogenes and large complex repeat structures. Also, the short average sequence length in the 111.6 Mb generated by the Roche/454 technology (100 nucleotides; Table 1) practically rules out a meaningful repeat analysis. We therefore concentrated on the mere identification of tandem repeats and transposon-derived interspersed repeats in the 5.4 Mb of both *N. furzeri *strains and *N. kunthae *generated by Sanger sequencing and, for comparison, analyzed our samples of medaka, stickleback, tetraodon and zebrafish genomes.

We considered as tandem repeats microsatellites, minisatellites and satellites composed of 1-5 nucleotides, 6-99 nucleotides, and over 100 nucleotides per repeat unit, respectively. About 1% of the *N. furzeri *DNA is composed of microsatellites, which is comparable with tetraodon (1.1%) and stickleback (0.8%), about half as much as in zebrafish (2%) and five times more than in medaka (0.2%) (Table 2). Roest Crollius *et al. *[31] reported that microsatellites comprise 3.21% of the tetraodon genome. The higher number is most probably due to the different algorithms and motif sizes applied; that is, Roest Crollius *et al. *used a Smith and Waterman algorithm-based approach previously applied to *Takifugu rubripes *[37] and a motif size of 1-6 nucleotides, while we used the program Sputnik [38], a specific tool for the detection of microsatellites, and a motif size of 1-5 nucleotides.

In *N. furzeri*, dinucleotide repeats are the most common type of repeat (37%; Additional data file 5) and cumulatively occupy the third largest amount of sequence compared to the other tandem repeats (Table 3). The repeat motif AC is the most frequent (26%), which is slightly less than in tetraodon and stickleback (30% each), and considerably more than in medaka (10%) and zebrafish (15%; Additional data file 5).

**Table 3:** Top 10 list of tandem repeats in the *N. furzeri *genome

Repeat unit* (bp)	G+C content^† ^(%)	Occupied sequence (bp)	Fraction of all tandem repeat sequences (%)	Fraction of genomic sequence (%)
77	63.6	511,132	45.84	9.53
348	41.1	339,743	30.47	6.33
2	ND	36,256	3.25	0.68
49	65.3	29,118	2.61	0.54
93	61.3	13,977	1.25	0.26
39	20.5	10,421	0.93	0.19
30	58.1	10,260	0.92	0.19
24	ND^‡^	10,018	0.90	0.19
4	ND	9,360	0.84	0.17
110	59.1	7,479	0.67	0.14

*Ranking according to occupied base pairs in 5.4 Mb of *N. furzeri *GRZ as given in column 3. ^†^For consensus sequence of one repeat unit, and deduced from sequences of most similar repeat units only. ^‡^The 24-nucleotide microsatellite comprises a heterogeneous fraction. The genomic clone used for FISH analysis has a G+C content of 58.1%. ND, not determined.

**Figure 4:**
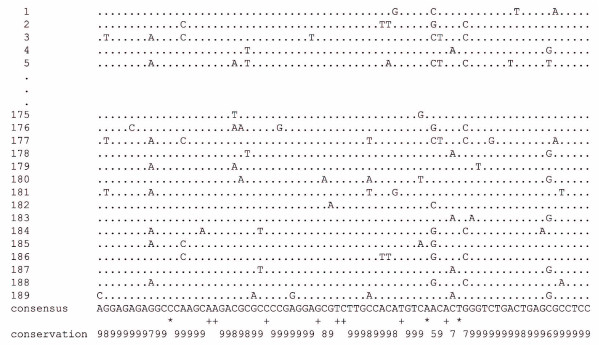
Sequence alignment of 189 monomers of the most abundant minisatellite of *N. furzeri*. The upper part shows a representative section of a ClustalW alignment of 189 monomers of the 77-nucleotide minisatellite of *N. furzeri *GRZ. Below, the deduced repeat consensus sequence and sequence variability are given based on all 189 monomers. Asterisks mark identical nucleotides, plus signs indicate one mismatch in 189 sequences. Numbers indicate nucleotide identities: 5 represents ≥50-60% identity for 189 sequences; 6 represents ≥60-70%; 7 represents ≥70-80%, 8 represents ≥80-90%; and 9 represents ≥90-100%.

**Figure 5:**
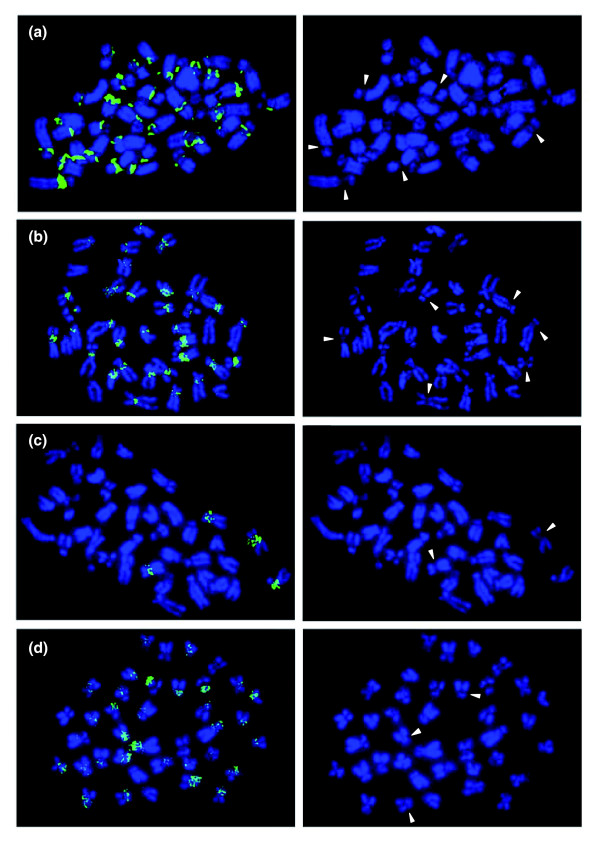
FISH analysis of the most frequent *N. furzeri *GRZ tandem repeats. **(a) **The most abundant, G+C-rich minisatellite, which is comprised of 77-nucleotide monomers, is found in centromeric regions of most chromosomes. **(b) **The second most abundant G+C-rich minisatellite, which is comprised of 49-nucleotide monomers, also forms centromeric regions of many chromosomes. **(c) **A G+C-rich, 24-nucleotide minisatellite specifically stains centromeric regions of two chromosome pairs. **(d) **The most frequent G+C poor satellite, which is comprised of 348-nucleotide monomers, maps to centromeric regions of many chromosomes. Panels on the right side show corresponding DAPI images to better illustrate the staining of centromeric regions. Arrows highlight selected distinct DAPI dull centromeric regions.

Minisatellites are far more abundant in *N. furzeri *than in the other four fish species. In particular, a 77-nucleotide minisatellite is most frequent. It comprises approximately 10% of the 5.4 Mb (Table 3) and its consensus sequence has a G+C content (63.6%) well above the genome average. Sequence conservation is high, for example, in an alignment of 189 repeat monomers; 65 of 77 positions (84%) are identical in at least 90% of monomers (Figure 4). Using *in situ *hybridization we found that this minisatellite localizes to centromeric regions of many chromosomes (Figure 5a). Also, the 77-nucleotide minisatellite is *N. furzeri *specific as we did not detect this or similar tandem repeats in available genomic sequences of other fishes and vertebrates. We identified several other abundant and G+C-rich minisatellites (Table 3), which also localize to centromeric regions. For example, a 49-nucleotide minisatellite is also found in centromeric regions of many chromosomes, whereas a 24-nucleotide minisatellite specifically localizes to centromeres of only two chromosomes (Figure 5b, c). Based on these analyses, we conclude that the large blocks of heterochromatin in centromeric regions, which are visualized at the cytological level by G+C-specific staining methods (Figure 1c), are mainly composed of *N. furzeri*-specific and abundant G+C-rich tandem repeats. We plan to isolate additional minisatellites to be used in a more elaborate, multi-color fluorescence *in situ *hybridization (FISH) study to assess chromosome specificity and derive chromosome-specific probes.

Hundreds of repetitions of the minisatellite motif 5'-TTAGGG-3' are found at telomeric ends of vertebrate chromosomes [39] and with associated proteins keep telomeres in homeostasis [40]. The telomeric repeat is not contained in the 5.4 Mb of *N. furzeri*. Most likely this is due to the limited sample size and so we searched for the repeat motif in the 111.6 Mb sample. We found 50 sequences, 14 of which are entirely composed of [TTAGGG]_n_. Correspondingly, in FISH experiments using the 5'-TTAGGG-3' motif as probe, specifically the terminal ends of chromosomes were labeled (I Nanda, unpublished results). Thus, characteristic vertebrate telomeric structures are present in *N. furzeri *and will be described elsewhere [15].

In addition to the G+C-rich minisatellites reported above, there is a prominent satellite repeat in *N. furzeri*. It comprises approximately 6% of our genomic sequence samples and has a monomer length of 348 nucleotides (Table 3). The G+C content of its consensus sequence (41.1%) is below the genome average. This satellite accounts for a second anomaly in the G+C distribution of *N. furzeri*; it causes an excess of sequences at approximately 41% G+C (Figure 3a). We found that this repeat also localizes to centromeric regions of many chromosomes (Figure 5d). It is currently not clear if higher order repeat structures are formed by the two types of tandem repeats and if these are specific to certain chromosomes, or if and how this impacts on the structural organization of the *N. furzeri *genome [41,42]. A direct influence on *N. furzeri *life span seems unlikely, since the tandem repeat content of the GRZ and MZM-0403 strains is identical while their life spans differ by a factor of two. It is tempting, however, to speculate that these tandem repeats play a role in cell division, and that both the exceptional size and composition of *N. furzeri *centromeres might be functionally linked to the extremely fast growth of young fish. In mammals, repetitive DNA becomes demethylated over age, which might affect chromosome structure in constitutive heterochromatin composed of such repeats. Since DNA methylation is also observed in fishes [43-46], it will be interesting to analyze whether methylation occurs in the G+C-rich tandem repeats of *N. furzeri *and whether methylation changes with age, or differs between strains exhibiting different life spans.

In summary, tandem repeats, comprising a total of 20.6% of the genome, are exceptionally abundant in *N. furzeri*. They are 4-12 times more frequent than in zebrafish (5.0%), tetraodon (3.6%), stickleback (2.1%) and medaka (1.7%; Table 2). In tetraodon, two major satellites are reported [31]. One is a subtelocentric, highly variable ten-nucleotide minisatellite, which is also the most prominent tandem repeat in this fish, while the other is a centromeric, somewhat variable 118-nucleotide satellite also found in *Takifugu rubripes*.

Interspersed transposon-derived repeats are mobile genetic elements and common to many eukaryotic genomes. Using RepeatMasker and Repbase Update, the reference library of vertebrate repeats [47], we found that 7-9% of the *N. furzeri *5.4 Mb samples is composed of known interspersed repeats (Table 2). Of these, approximately 6.3% are retrotransposons and approximately 1.5% DNA transposons. To identify novel transposon-derived repeats, we used the *ab initio *repeat identification program RepeatScout [48], which was recently found to be suitable for analyzing short sequences, although originally developed to analyze longer sequences [49]. Accordingly, 16-18% of our *N. furzeri *genomic samples are composed of novel interspersed repeats. Unfortunately, a detailed classification of the novel repeats is currently not feasible, again due to the limited/fragmented sample size. However, we assume that the fraction of these repeats identified in the 5.4 Mb closely resembles the overall fraction present in the *N. furzeri *genome, because our estimate for the medaka genomic sample corresponds well with the estimate given for the medaka draft genome [20]. In total, interspersed repeats comprise 24.7% of the *N. furzeri *genome, which isconsiderably more than in tetraodon (3.4%), stickleback (4.5%), and medaka (13.6%), and less than in zebrafish (35.4%). The overall repeat content in *N. furzeri *thus amounts to approximately 45%, which is the highest among the analyzed fish species. We note that this value might still be an underestimate, as we can not exclude that the interspersed repeat content might be found to be higher upon analysis of larger genomic samples since it is conceivable that we have missed 'rare' repeats in our samples.

### Protein coding sequences

We attempted to explore the protein coding fraction contained in the 5.4 Mb of *N. furzeri *GRZ genomic sequence with respect to conservation in medaka, stickleback, tetraodon, zebrafish and human. As indicated above, we found that 444 of the *N. furzeri *GRZ sequences (473 kb of the 5.4 Mb) bear fragments of protein coding genes (Additional data file 2). Roughly one-third (152 kb) of the sequences are actually coding, and most of these (399, 94.3%) contain one to three exons. The G+C content of the 444 sequences (44.1%) fits the genome average and is considerably higher (50.3%) in the coding portions. Similar observations were made in tetraodon and medaka [19,20].

Of the 444 sequences, 310 (70%) show best matches to fishes, while 32 (7%) and 23 (5%) match best to human and mouse, respectively (Additional data file 2). Furthermore, 410 (92%) of the 444 *N. furzeri *gene fragments have homologs in at least one of the other four fish species with amino acid identities of 22-100%. For a subset of 180 (40%) *N. furzeri *gene fragments we identified homologs in all four fish species. In those, amino acid conservation is highest in medaka (77.4%) and stickleback (77.1%) followed by tetraodon (75.2%) and zebrafish (70.1%). Lastly, of the 34 (8%) *N. furzeri *gene fragments without a counterpart in currently available sequences of the four fish species, four match best to other fish species (58-100% amino acid identity), 13 to mammalian/vertebrate proteins (26-67% amino acid identity), and 17 to plant, fungal, invertebrate and other proteins (27-72% amino acid identity) (Additional data file 6).

In a complementary approach, we cloned complete coding sequences of five *N. furzeri *genes, *Cdkn2b*, *Cdkn2d*, *Msra*, *Sirt1 *and *Tp53*. Their gene products are involved in different aspects of aging and functionally conserved across taxa [1,50,51]. Sirtuin1 is a NAD(+)-dependent histone deacetylase considered to mediate the life span extending effects of calorie restriction [52], and its aging regulating function is conserved from yeast to mammals. Msra repairs and protects proteins from oxidation [53], and mice with mutations in it show a reduced life span [54]. Cdkn2b, Cdkn2d and Tp53 control cell cycle progression and mediate senescence [55-57]. A comparison of respective *N. furzeri *and human proteins should give an indication as to the potential use of this fish as a model for human biology. The five *N. furzeri *proteins are 40-65% conserved in humans (Table 4), which is in the range reported for orthologous proteins of teleost fishes and humans (60-70%) [19]. For comparison with zebrafish, which is perhaps the best established fish model for human biomedical phenotypes [58], we deduced zebrafish *Cdkn2b*, *Cdkn2d*, *Msra*, *Sirt1 *and *Tp53 *genes based on database searches and alignments of expressed sequence tags to genomic DNA (Additional data file 7). Conservation of inferred zebrafish proteins to human orthologs is in the same range (41-65%) as for *N. furzeri *and humans (Cdkn2b, 41%; Msra, 65.3%; Sirt1, 48.9%; Tp53, 48%). Conservation of the five *N. furzeri *aging relevant genes with respect to the other four fish species is highest in medaka, followed by stickleback, tetraodon and zebrafish (Table 4), the same as for the 180 random gene fragments described above.

**Table 4:** Comparison of five aging relevant genes of *N. furzeri *with orthologs of medaka, stickleback, tetraodon, zebrafish and humans

Gene*	*N. furzeri GRZ *(amino acids)	Medaka (%)^†^	Stickleback (%)^†^	Tetraodon (%)^†^	Zebrafish (%)^†^	Human (%)^†^
*Cdkn2b*	128	NA	NA	66.4	52.3	49.3
*Cdkn2d*	165	89.1	84.2	79.0	NA	53.0
*Msra*	237	78.0	75.3	79.0	74.5	64.7
*Sirt1*	689	72.0	71.8	69.4	53.8	51.8
*Tp53*	372	59.1^‡^	56.6	59.1	45.5	40.0

*Sequences used for alignments are given in Additional data file 7. ^†^Protein identity is given. ^‡^Partial sequence. NA, not available.

Finally, we attempted to relate the 444 gene fragments and 5 aging related genes with respect to conservation in humans. We analyzed exons encompassing at least 33 amino acids, which number 366 in the former data set and 21 in the latter. In these exons, conservation with human counterparts is considerably higher for random gene fragments than for aging related genes: 67% versus 62%. Currently, we can only speculate about the reasons for this difference. One possibility might be that it reflects a bias introduced by the sampling procedure of the 444 gene fragments. On the other hand, the difference might be related to the huge difference in life span between *N. furzeri *and human.

### Phylogeny

**Figure 6:**
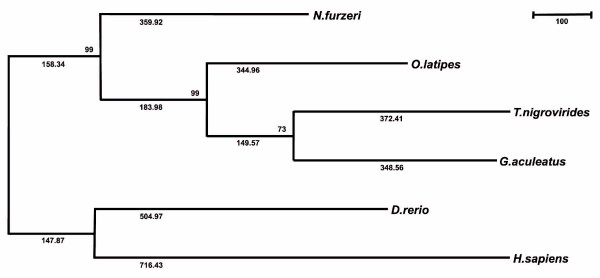
Evolutionary relationships of *N. furzeri *to medaka, stickleback, tetraodon, zebrafish and human. The evolutionary history based on the maximum parsimony (MP) method [79] and MEGA4 [78] is shown and the most parsimonious tree given (length = 3,287). The tree topology is fully consistent with the taxonomic classification of the analyzed fish species. Accordingly, *Nothobranchius *is most closely related to medaka, while both zebrafish and human are distantly related to the euteleost fish division.

In an initial attempt at phylogenetic reconstruction, we used the *N. furzeri *28S rRNA gene (Additional data file 8) in comparison with 15 fish and two tetrapod sequences. However, standard parsimony analysis resulted in tree topologies much different from widely accepted taxonomic groupings (data not shown), indicating that the phylogenetic information contained in the 28S rRNA locus does not resolve the evolutionary history of fishes and tetrapods. We thus attempted to infer the phylogenetic positioning of *N. furzeri *with respect to the four fish model species and humans based on the 444 partial protein coding sequences and aging related genes identified in this work. A phylogenetic tree obtained by the maximum parsimony approach (Figure 6) is fully consistent with the taxonomic classification of the analyzed fish species. Accordingly, *Nothobranchius *is most closely related to medaka. Zebrafish and humans are both distantly related to the euteleost fish division consisting of the Atherinomorpha *Nothobranchius *and medaka on the one hand, and the Percomorpha stickleback and tetraodon on the other. The same tree topology was obtained using the neighbor-joining method, giving slightly lower bootstrap confidence (data not shown). We conclude that the coding fragments are sufficient to create a stable phylogeny, as previously suggested [59]. Our data are in agreement with phylogenetic analyses performed for ten fish species, which, based on expressed sequence tag analyses, found both killifish and medaka to be more distant from pufferfish than from each other [59].

### Genetic variability

The *N. furzeri *GRZ strain studied in this work most probably originates from a single pair, which stems from wild fish captured in 1968, and was used for breeding more than 30 years ago [11]. If we assume a generation time of 6 months - comprising 12-16 weeks following hatching and about the same time for embryonic development - GRZ fish would have been inbred for approximately 80 generations until today. Thus, it was generally assumed that GRZ fish are highly homogeneous [17], although experimental proof was lacking.

To assess the genetic variability of the GRZ strain, we genotyped eight microsatellites (four di-, three tri- and one tetranucleotide repeat, identified in the 5.4 Mb), in 20 GRZ specimens. All microsatellites are monomorphic; we observed only one allele in the 20 specimens, respectively (Additional data file 9). To extend the analysis to gene associated markers, we resequenced parts of 19 protein coding genes encompassing 4 exonic and 15 intronic regions (10.2 kb in total). Again, there was no sequence variation in the 20 GRZ fish. In a third approach, we performed multi-locus fingerprinting in ten GRZ specimens using the 5'-GGAT-3' repeat as a marker [60]. As for the other markers, all specimens were homozygous at all 5'-GGAT-3' marker loci. Thus, we provide here the first experimental confirmation that the GRZ strain is highly inbred. In contrast, *N. furzeri *strain MZM-0403, bred in the sixth captive generation in our facility, exhibits 2-6 alleles in the microsatellites and has single nucleotide variations in 14 of the 19 genes. Also, a laboratory strain of the closely related species *N. kunthae *is highly heterogeneous in all markers that could be analyzed (Additional data files 9 and 10). While the inbreeding status of the GRZ strain is advantageous for further genetic analyses, it is currently unclear to what extent its short life span results from inbreeding depression [61]. A reduction of fitness can be caused by the accumulation of deleterious alleles [62], and reduced genetic diversity often affects birth weight, survival, reproduction and resistance to disease [63]. In this respect is noteworthy that the *N. furzeri *GRZ strain is derived from one 'normal' breeding pair that, 35 years ago, lived "a good 4 and a half months" (16-18 weeks), and that "healthy young fish" were used for spawning [11]. In our facility, the 10% survivorship (that is, the maximum life span) of direct descendants of these fish is 16 weeks and the fish can live up to 21 weeks [15], which is well in the range reported for the founders. Furthermore, there are differences in age-related histological damage between GRZ and age matched MZM-0403, which argue for accelerated aging in the GRZ strain rather than inbreeding-related early death. For example, measurement of lipofuscin, which is an autofluorescing pigment that accumulates over age in a number of species [64], shows accelerated aging in brain and liver of the GRZ strain compared to age matched MZM-0403 [18]. To assess the degree of inbreeding depression on the life span of *N. furzeri *GRZ, access to new *N. furzeri *wild isolates from the GRZ game reserve in Zimbabwe or a nearby location is highly desirable. These fish could be cross-bred with the inbred GRZ strain and traits related to fitness, such as growth rate, reproduction, and survival, could be recorded and compared to the parental GRZ strain/wild isolate. On the other hand, it has to be noted that inbred lines are extremely powerful tools for biomedical research; if not subjected to severe inbreeding depression, they are valuable in many respects as they provide a uniform and stable genetic background and respective phenotype(s). Our results prove the feasibility of establishing *N. furzeri *inbred lines, which in the future may become instrumental in dissecting complex traits like life span.

## Conclusion

Our analyses provide a first glimpse into the genome of the short-lived annual fish *N. furzeri*, which we would like to support as a new vertebrate model for age research, as has been suggested previously [9,12,17].

At an estimated 1.6-1.9 Gb, *N. furzeri *has the largest genome compared with medaka, stickleback, tetraodon and zebrafish, which serve as models in many areas of contemporary research and for which genome projects have been completed or are underway. At the cytological level, a compartmentalization of the *N. furzeri *genome is apparent, which is caused by an accumulation of G+C-rich heterochromatin in centromeric regions of most chromosomes. This is mirrored in the composition of *N. furzeri *repeats, which among the analyzed fishes occupy the largest genomic fraction and are distinguished by an unmatched portion of G+C-rich and -poor tandem repeats. The most abundant G+C-rich species-specific minisatellites localize to centromeric regions and cause an unusual second peak in the G+C content distribution of the *N. furzeri *genome. The exceptionally high tandem repeat and G+C content is neither an inbreeding artifact of the GRZ strain nor can it be directly linked to life span, since genome structure and repeat content do not differ between the *N. furzeri *GRZ and MZM-0403 strains, although the former is highly inbred and the latter is a recently wild-derived strain and they differ in life span by a factor of two. The high tandem repeat and G+C content rather represent an *N. furzeri*-specific feature since this is much less pronounced in the closely related species *N. kunthae*, and has so far not been observed in vertebrates.

The *N. furzeri *and *N. kunthae *genomic sequences are a source of markers that can be used to build a first generation linkage map as well as to assess the genetic variability of *Nothobranchius *laboratory strains and natural populations with different life spans. An initial marker set provided the first molecular confirmation that the *N. furzeri *strain GRZ is highly inbred in contrast to the recently wild-derived strain MZM-0403 as well as the *N. kunthae *laboratory strain, which were found to be very heterogeneous.

Cross-breeding of *N. furzeri *strains with different life spans is currently being performed in our laboratory and should enable the identification of quantitative trait loci and facilitate cloning of aging-relevant genetic determinants. The present study illustrates the challenges that will have to be addressed in an *N. furzeri *genome project that we would like to establish in order to make maximal use of this fish species as a vertebrate model for aging research.

## Materials and methods

### Specimens

For genomic sequencing, *N. furzeri *GRZ obtained in 2002 from Marc Bellemans (Hove, Belgium) were used. Karyotyping and chromosome stainings were done using: two male and female *N. furzeri *GRZ specimens obtained from Werner Krammer (Deutsche Killifisch Gesellschaft, Ausgburg, Germany); one male *N. orthonotus *from Nisa (Mozambique) collected by MaS and SS in 2006; one male *N. hengstleri *collected in 2005 in Mozambique from location MZHL-2005-14; and one male *N. eggersi *collected in 1997 in Tanzania from location TZ 97/55. Fish were kept in 40 liter tanks at 26°C and room temperature with sponge filters driven by an air supply. Water changes were performed every other day.

### Chromosome preparations and banding analyses

To obtain mitotic chromosomes, healthy fish were exposed to a 0.03% colchicine solution for 8-12 h in a well ventilated container. Then fish were sedated on ice, killed by caput dislocation, and gills, spleen and kidney carefully removed. Preparation of cell suspension, hypotonic treatment, fixation of cells, and preparation of slides were as described [65]. Conventional chromosome staining, fluorescence staining with DAPI (4',6-diamidino-2-phenylindole) and mithramycin were as described [66]. To enhance the mithramycin fluorescence, slides were first pre-incubated with distamycin A. The location of constitutive heterochromatin on chromosomes was visualized as reported [67].

### Flow cytometry measurements

Fin clips were taken from three *N. furzeri *GRZ females and at least 10,000 cells per sample measured on a Cell Analyzer CAII (Partec, Muenster, Germany). As reference, similar numbers of erythrocytes of a female chicken were used. Preparation of cell suspensions, fixation, DAPI staining and measurements were as described [28]. PI staining was performed at a final concentration of 50 μg/ml for 30 minutes at 37°C as described [60]. For each dye, three independent measurements were performed.

### Fluorescence *in situ *hybridization

FISH mapping of repetitive DNA on *N. furzeri *GRZ chromosomes was performed as described [68]. Briefly, a biotin labeled plasmid clone bearing a specific tandem repeat was denatured and hybridized overnight at 37°C to denatured metaphase chromosomes. After post-hybridization washes at low (2× SSC, 50°C) and moderately (1× SSC, 60°C) stringent conditions, hybridization sites were detected with fuorescein isothiocyanate (FITC) conjugated avidin (Vector Laboratories, Burlingame, CA, USA) followed by signal amplification through incubation of slides with biotinylated anti-avidin and FITC conjugated avidin. Slides were examined on a microscope with a CCD camera (Zeiss, Goettingen, Germany); chromosomes showing specific FITC signals were displayed on counter-stained DAPI metaphases using appropriate software (Applied Spectral Imaging, Neckarhausen, Germany).

### DNA/RNA

For genomic library construction, DNA of one male specimen of each of *N. furzeri *GRZ, *N. furzeri *MZM-0403, and *N. kunthae*, was isolated from 30 mg frozen tissue with the Blood and Cell Culture DNA Mini kit (Qiagen, Hilden, Germany). For marker validation, genomic DNA from 20 *N. furzeri *GRZ, 10 MZM-0403 and 10 *N. kunthae *specimens was isolated using 30 mg frozen tissue and AquaGenomicSolution (MobiTec, Goettingen, Germany), respectively. For GRZ, 50 ng DNA from ten specimens each were combined in a pool. For cloning of known aging related genes, RNA was isolated from one *N. furzeri *GRZ male using the RNeasy Mini kit (Qiagen). Primer sequences are given in Additional data file 11.

### Sequencing

For shotgun sequencing using Sanger technology, randomly sheared, end-repaired 1-3 kb DNA fragments were ligated into pUC18. Recombinant plasmids were amplified in *E. coli*, purified, sequenced from both ends (BigDye Terminator v.1.1 Cycle Sequencing Kit, ABI, Weiterstadt, Germany) and separated on ABI 3730xl capillary sequencers. After quality clipping, sequences were assembled based on overlaps and read pair information [69]. Contaminations were identified by BLASTN searches in GenBank sections bacteria, protozoa and phage (version 161, 15.08.2007 [70]) and removed. Then, a database comprising all sequences was built and each sequence searched against the database using WU-BLASTN. A sequence was regarded redundant if: in addition to the best hit against itself another hit was found (*p *≤ 10^-30^, ≥95% identitiy); and no other matches were found. Redundant sequences were excluded. Remaining data comprise 5,540 contigs and 5.36 Mb for *N. furzeri *GRZ, 5,686 contigs and 5.39 Mb for *N. furzeri *MZM-0403, 6,273 contigs and 5.36 Mb for *N. kunthae *(Table 1). Contigs are referred to as sequences in the text. All Sanger sequences were submitted to the National Center for Biotechnology Information (NCBI). Also, a database was created and is available for BLAST searches at [71]. For genomic sequencing using Roche/454 Life Sciences GS20 technology, 5 μg of *N. furzeri *GRZ genomic DNA were used and 111.6 Mb sequenced (Table 1). These sequences were not assembled prior to analyses and submitted to the trace archive at NCBI.

### PCR

Primers were designed using the GAP4 module of the Staden Sequence Analysis Package [72] and ordered from Metabion (Martinsried, Germany). For PCR, 50 ng DNA, 10 pmol of each primer and PuReTaq Ready-To-Go PCR beads (GE Healthcare, Munich, Germany) were used; initial denaturation was at 94°C for 30 s followed by 35 cycles of 60 s at 94°C, 60 s at primer annealing temperature, 60 s extension at 72°C, and a final extension at 72°C for 300 s. PCR products were sequenced as described above. Sequences were visualized, edited and assembled in GAP4.

### Marker generation and experimental validation

Microsatellites were identified in the 5.4 Mb of *N. furzeri *GRZ using Sputnik (see Repeat identification). Flanking primers were designed in sequences with: one microsatellite; at least 20 repeat units for dinucleotide repeats, or at least 10 repeat units for tri- and tetranucleotide repeats; at least 100 nucleotides of flanking sequence; perfect repeats. PCR was performed in two pools of GRZ DNA (see DNA/RNA) with one primer being 6-FAM-labeled. For genotyping, ABI 3730xl sequencers and GeneMapper software Version 4 was used. Allele calling was performed independently by two individuals. Discrepancies were resolved by retyping. For resequencing of gene-associated markers, primers were designed in 19 sequences with significant homology to known genes in at least one of the four fish reference genomes. PCR and sequencing was performed in two DNA pools of GRZ, and ten individual *N. kunthae *fish as described above.

### Reference data sets

In order to compare our genomic data of *N. furzeri *strains GRZ and MZM-0403, and *N. kunthae*, which we obtained by random genome-wide sample sequencing, we simulated this approach *in silico *for the other four fish species. That is, we extracted random genomic data from the publicly available genome assemblies for each. In detail, these were 20 samples of random genomic sequence sets of medaka, stickleback, tetraodon and zebrafish from genome assemblies at *Ensembl *[21] (*Tetraodon nigroviridis *update release (2007-05-29), *Gasterosteus aculeatus *update release (2007-05-30), *Oryzias latipes *update release (2007-05-24), *Danio rerio *update release (2007-07-30)). For ten samples, each reference data set represented 0.4% of the respective genome and had a sequence length distribution corresponding to the 5.4 Mb of *N. furzeri *generated by Sanger sequencing. For the other ten samples, sequence sets comprised 6% of the respective genome with a length distribution corresponding to the 111.6 Mb of *N. furzeri *GRZ sequenced by 454/GS20 technology. For genome size estimation, random genomic sequences of medaka, stickleback, tetraodon, and zebrafish were extracted in the same way; each comprised 5.4 Mb and had a sequence length distribution similar to the *N. furzeri *5.4 Mb.

### BLAST analyses

To determine the coding fraction, a BLASTX search in Swiss-Prot/TrEMBL (version 50.6) was performed. Hits with *p *< 10^-10 ^were regarded significant. Reference data sets of the four fish species were analyzed in the same way. Then, *N. furzeri *GRZ and MZM-0403 sequences containing coding portions were used for TBLASTX searches in medaka, stickleback, tetraodon and zebrafish cDNA collections downloaded from *Ensembl *[21] (see 'Reference data sets' above for release dates). A *p*-value < 10^-10 ^was set as the threshold for significance.

### Repeat identification

First, tandem repeats were identified and masked (replaced by Ns) using Tandem Repeats Finder, version 4.00 [73] with the following parameters: Match 2, Mismatch 7, Delta 7, PM 80, PI 10, Minscore 50, MaxPeriod 1,300 (half the length of the longest sequence in the *N. furzeri *5.4 Mb). Second, known interspersed repeats - that is, sequences matching entries from Repbase Update [47] - were identified and masked using RepeatMasker (version open-3.1.8; AFA Smit, R Hubley and P Green, unpublished data) [74], with standard parameters. Third, for *de novo *repeat identification, RepeatScout, version 1.0.2 [48] with standard parameters, except for a minimum repeat copy number of 3, was used on the pre-masked sequences. Finally, a species specific repeat consensus library was built using both the identified known repeat families and the previously unknown repeat consensus sequences.

To detect microsatellites (1-5 nucleotides) the program Sputnik [38] with parameters -v 1 -u 5 was used.

### Phylogenetic analyses

Of the 444 gene fragments of *N. furzeri *GRZ, we first identified 44 for which we could unambiguously assign human orthologs based on BLASTX searches in Swiss-Prot. Based on these assignments, we searched for orthologous regions in medaka, stickleback, tetraodon, and zebrafish genome assemblies at University of California Santa Cruz (UCSC) [75] (oryLat1, tetNig1, gasAcu1, danRer5, hg18) by inspection of MAF tracks. We extracted exons, which we regarded as true orthologs if: no duplicated gene was detected in the respective species; splice sites were conserved; and no frameshifts were detected if consecutive exons were concatenated. In total, this resulted in 71 orthologous exons, identified in all six species (average length is 142 bp for human exons), which represent fragments of 26 protein coding genes (sequences are listed in Additional data file 12, including exons of *N. furzeri *GRZ). Five of the gene fragments represent one exon, while the others span at least one splice site. For further analyses, sequences were concatenated, translated, aligned by ClustalW v1.81 with standard parameters [76] and trimmed at the ends if necessary. Coding sequences of the aging related genes *Msra*, *Sirt1*, and *Tp53 *were also translated and aligned. All alignments were concatenated, resulting in 4,828 amino acid positions, and analyzed by MEGA4 [77] with different methods (alignment available on request). Positions containing gaps and missing data were eliminated, leaving a total of 4,245 positions in the final dataset, of which 508 were parsimony informative. A maximum parsimony tree was obtained using the Close-Neighbor-Interchange algorithm in which the initial trees were obtained with the random addition of sequences (100 replicates).

### Sequence comparison

Nucleotide sequences of putative orthologs of the four analyzed fish species were identified as described above and are listed in Additional data file 7. Nucleotide and translated sequences were aligned using needle (EMBOSS, version 3.0.0 [78]).

### Database entries included in this work

The 5.4 Mb of *N. furzeri *GRZ genomic DNA sequence generated by the Sanger technique have been deposited under the project accession [GenBank:ABLO00000000]. The version described in this paper is the first version ABLO01000000. The 111.6 Mb of *N. furzeri *GRZ genomic sequence generated by Roche/454 technology have been deposited at GenBank under project id (PID) 29535. The 5.4 Mb of *N. furzeri *MZM-0403 genomic DNA sequence generated by the Sanger technique have been deposited under the project accession [GenBank:ACCZO00000000]. The version described in this paper is the first version [GenBank:ACCZ01000000]. The 5.4 Mb of *N. kunthae *genomic sequence generated by the Sanger technique have been deposited under the project accession [GenBank:ACDA00000000]. The version described in this paper is the first version [GenBank:ACDA01000000]. *N. furzeri *GRZ major rRNA gene cluster [GenBank:EU780557]; *Cdkn2b *[GenBank: EU271680]; *Cdkn2d *[GenBank: EU400615]; *Msra *[GenBank EU400617]; *Sirt1 *[GenBank:EU271679]; *Tp53 *[GenBank:EU271681].

## Abbreviations

DAPI: 4',6-diamidino-2-phenylindole; FISH: fluorescence *in situ *hybridization; FITC: fluorescein isothiocyanate; GRZ: Gona Re Zhou; NCBI: National Center for Biotechnology Information; PI: propidium iodide.

## Authors' contributions

CE and MP initiated the project. KR coordinated the project, and generated and assembled most sequences. ST and MBS helped with sequencing *N. furzeri *GRZ. AC and MaS provided biological specimens. KR, CL, and MP analyzed the sequences. JK constructed the genomic library of *N. kunthae *and performed genotyping. NH cloned aging related genes. IN, SS, MiS and MaS performed cytogenetic studies and genome size estimation by flow cytometry. GG helped with the genome size estimation based on sequences. UG and KS performed phylogenetic analyses. KR, UG, KS, MaS, AC, CE and MP contributed to writing the manuscript. All authors read and approved the final manuscript.

## Additional data files

The following additional data are available with the online version of this paper. Additional data file 1 is a table showing the genome size estimation for *N. furzeri *based on sequence data. Additional data file 2 is a table listing *N. furzeri *GRZ gene fragments identified by BLASTX searches in Swiss-Prot/TrEMBL. Additional data file 3 is a figure showing flow cytometry measurements to estimate the *N. furzeri *genome size. Additional data file 4 is a figure of G+C histograms of *N. furzeri *strain MZM-0403 and the closely related species *N. kunthae*. Additional data file 5 is a figure showing microsatellite frequencies of *N. furzeri *GRZ. Additional data file 6 is a table listing the subgroup of *N. furzeri *GRZ gene fragments lacking homologs in medaka, stickleback, tetraodon, and zebrafish. Additional data file 7 is a table that lists chromosomal locations and accession numbers of sequences used for analyses of aging related genes. Additional data file 8 is a table showing the conservation of the *N. furzeri *GRZ major rRNA cluster in tetraodon and human. Additional data file 9 is a table showing genotypes of microsatellites analyzed in *N. furzeri *strains GRZ and MZM-0403 and the closely related species *N. kunthae*. Additional data file 10 is table showing the analysis of 19 gene-associated markers in *N. furzeri *strains GRZ and MZM-0403 and *N. kunthae*. Additional data file 11 is a list of primers used to clone and sequence five aging related genes in *N. furzeri *GRZ. Additional data file 12 is a table showing nucleotide and amino acid sequences used for phylogenetic analyses.

## Supplementary Material

Additional data file 1 Coding sequences identified in random genomic sequence samples of medaka, stickleback, tetraodon and zebrafish, their genome sizes, and inferred *N. furzeri *genome size are given. Click here for file

Additional data file 2 *N. furzeri *GRZ gene fragments identified by BLASTX searches in Swiss-Prot/TrEMBL. Click here for file

Additional data file 3 Flow cytometry measurements to estimate the *N. furzeri *genome size. Click here for file

Additional data file 4 G+C histograms of *N. furzeri *strain MZM-0403 and the closely related species *N. kunthae*. Click here for file

Additional data file 5 Microsatellite frequencies of *N. furzeri *GRZ. Click here for file

Additional data file 6 The subgroup of *N. furzeri *GRZ gene fragments lacking homologs in medaka, stickleback, tetraodon, and zebrafish. Click here for file

Additional data file 7 Chromosomal locations and accession numbers of sequences used for analyses of aging related genes. Click here for file

Additional data file 8 Conservation of the *N. furzeri *GRZ major rRNA cluster in tetraodon and human. Click here for file

Additional data file 9 Genotypes of microsatellites analyzed in *N. furzeri *strains GRZ and MZM-0403 and the closely related species *N. kunthae*. Click here for file

Additional data file 10 Analysis of 19 gene-associated markers in *N. furzeri *strains GRZ and MZM-0403 and *N. kunthae*. Click here for file

Additional data file 11 Primers used to clone and sequence five aging related genes in *N. furzeri *GRZ. Click here for file

Additional data file 12 Nucleotide and amino acid sequences used for phylogenetic analyses. Click here for file
