# Supplementary material for: High tandem repeat content in the genome of the short-lived annual fish Nothobranchius furzeri: a new vertebrate model for aging research
Source: Genome Biol. 2009 Feb 11;10(2):R16. doi: 10.1186/gb-2009-10-2-r16 (PMC2688266; doi:10.1186/gb-2009-10-2-r16)
Supplement: Additional data file 1 — Coding sequences identified in random genomic sequence samples of medaka, stickleback, tetraodon and zebrafish, their genome sizes, and inferred N. furzeri genome size are given. [file gb-2009-10-2-r16-S1.doc]

**Additional data file 1: Sequence based estimation of *N. furzeri genome size***

|  | genome  size  [Mb] | queries^1^ | hits in  Swiss-Prot/ TrEMBL^2^ | No. of coding sequences [%] | genome size estimate  for *N. furzeri* [Mb] |
| --- | --- | --- | --- | --- | --- |
|  |  |  |  |  |  |
| ***N. furzeri GRZ*** | tbd^3^ | 5540 | 444 | 7.9 |  |
| ***N. furzeri MZM-0403*** | tbd^3^ | 5686 | 443 | 7.8 |  |
| tetraodon^4^ | 402 | 5577 | 1739 | 31.2 | 1,588 |
| stickleback^5^ | 680 | 5496 | 1079 | 19.6 | 1,687 |
| medaka^6^ | 1,060 | 5795 | 749 | 12.9 | 1,731 |
| zebrafish^7^ | 1,441 | 5417 | 571 | 10.5 | 1,915 |
|  |  |  |  |  | **mean: 1,730** |
|  |  |  |  |  |  |

^1^ number of sequence contigs of respective genomic sample

^2^ hits to bacteria, virus and parasite DNA, repetitive elements, and mitochondrial DNA are excluded

^3^ tbd: to be determined

^4^ genome size according to the ‘reference assembly “Golden path” length of Tetraodon7, April 2003’ given at *Ensembl* (www.ensembl.org/Tetraodon_nigroviridis/index.html)

^5^ genome size according to David Kingsley (personal communication)

^6^ genome size according to Kasahara et al. (2007)

^7^ genome size according to the ‘reference assembly “Golden path” length” of Zv7, April 2007’ given at *Ensembl* (www.ensembl.org/Danio_rerio/index.html)
