# Supplementary material for: High tandem repeat content in the genome of the short-lived annual fish Nothobranchius furzeri: a new vertebrate model for aging research
Source: Genome Biol. 2009 Feb 11;10(2):R16. doi: 10.1186/gb-2009-10-2-r16 (PMC2688266; doi:10.1186/gb-2009-10-2-r16)
Supplement: Additional data file 3 — Flow cytometry measurements to estimate the N. furzeri genome size. [file gb-2009-10-2-r16-S3.doc]

## Additional data file 3: Genome size estimation of *N. furzeri* by flow cytometry

Flow histograms of dorsal fin cells of (A) female *N. furzeri* GRZ and (B) female chicken erythrocytes using conventional DAPI staining. The measured fluorescence intensity of the *N. furzeri* GRZ cells overlaps with the chicken reference peak. (C) Flow histogram of dorsal fin cells of female *N. furzeri* GRZ and reference chicken erythrocytes stained with PI.

**A**

**B**

**C**
