# Supplementary material for: High tandem repeat content in the genome of the short-lived annual fish Nothobranchius furzeri: a new vertebrate model for aging research
Source: Genome Biol. 2009 Feb 11;10(2):R16. doi: 10.1186/gb-2009-10-2-r16 (PMC2688266; doi:10.1186/gb-2009-10-2-r16)
Supplement: Additional data file 4 — G+C histograms of N. furzeri strain MZM-0403 and the closely related species N. kunthae. [file gb-2009-10-2-r16-S4.doc]

## Additional data file 4: G+C content distribution of *N. furzeri* MZM‑0403 and the

## closely related species *N. kunthae*

(A) Histogram of the G+C content of the 5.4 Mb genomic sample of *N. furzeri* MZM‑0403. The average G+C content is 44.3%. Note G+C distortions which are diagramed in a second peak at ~62% G+C and an unusually high number of sequences with ~41% G+C. A similar G+C content distribution is displayed in *N. furzeri* GRZ (see main manuscript).

(B) Histogram of the G+C content of the 5.4 Mb genomic sample of *N. kunthae*. The average G+C content is 44.9%. Note the absense of highly abundant G+C rich and G+C poor sequences as compared to both *N. furzeri* strain GRZ and *N. furzeri* strain MZM‑0403.


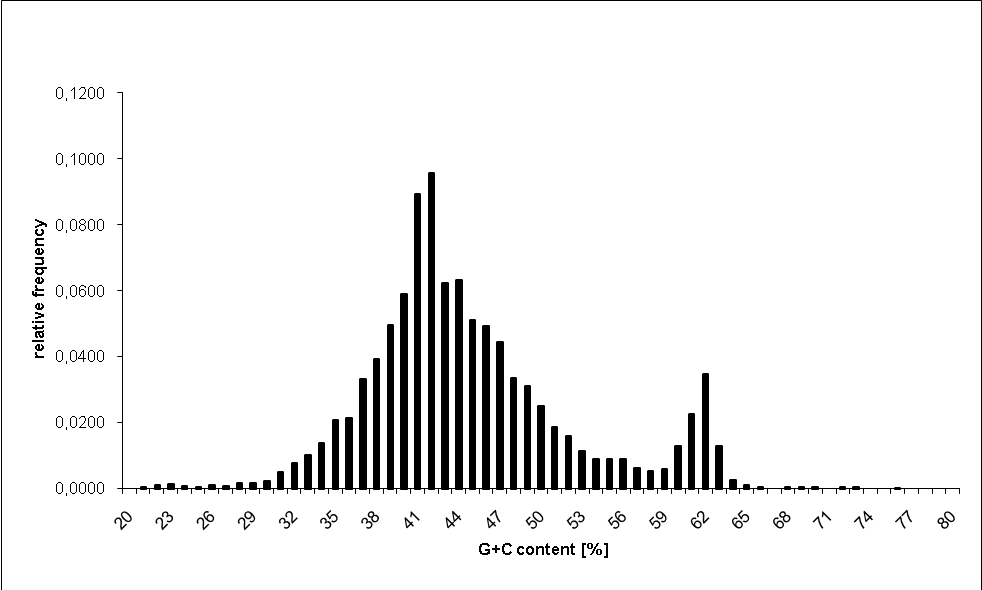


**A**


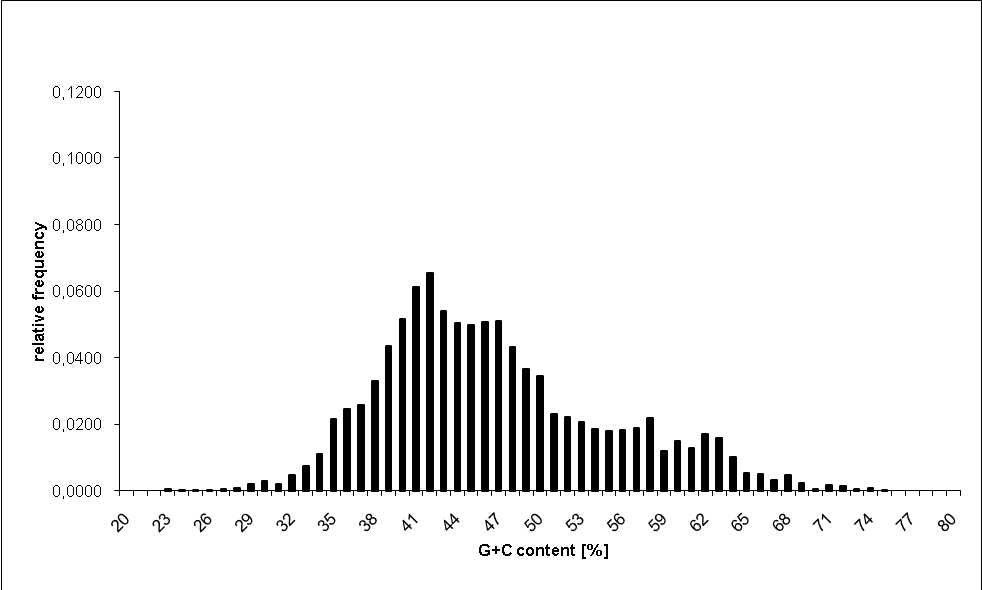


**B**
