# Supplementary material for: High tandem repeat content in the genome of the short-lived annual fish Nothobranchius furzeri: a new vertebrate model for aging research
Source: Genome Biol. 2009 Feb 11;10(2):R16. doi: 10.1186/gb-2009-10-2-r16 (PMC2688266; doi:10.1186/gb-2009-10-2-r16)
Supplement: Additional data file 5 — Microsatellite frequencies of N. furzeri GRZ. [file gb-2009-10-2-r16-S5.doc]

## Additional data file 5: Microsatellite frequencies of *N. furzeri*

Microsatellite frequencies were determined in the 5.4 Mb genomic sample of *N. furzeri* strain GRZ. (A) Relative frequencies of mono-, di-, tri-, tetra- and pentanucleotide repeats and (B) relative frequencies of eight most common repeat motifs in medaka, stickleback*,* tetraodon and zebrafish are shown.

**B**

**A**
