# Supplementary material for: High tandem repeat content in the genome of the short-lived annual fish Nothobranchius furzeri: a new vertebrate model for aging research
Source: Genome Biol. 2009 Feb 11;10(2):R16. doi: 10.1186/gb-2009-10-2-r16 (PMC2688266; doi:10.1186/gb-2009-10-2-r16)
Supplement: Additional data file 8 — Conservation of the N. furzeri GRZ major rRNA cluster in tetraodon and human. [file gb-2009-10-2-r16-S8.doc]

**Additional data file 8: Sequence comparison of *N. furzeri*, tetraodon, and**

**human major rRNA gene clusters**

|  |  |  |  |  |  |  |
| --- | --- | --- | --- | --- | --- | --- |
|  |  | **18S rRNA** | **ITS1** | **5.8S rRNA** | **ITS2** | **28S rRNA** |
|  |  |  |  |  |  |  |
|  |  |  |  |  |  |  |
| *N. furzeri GRZ* | length [bp] | 1,838 | 1,411 | 161 | 624 | 4,024 |
|  |  |  |  |  |  |  |
| tetraodon | length [bp] | 1,865 | 460 | 159 | 579 | 4,253 |
|  | identity to *N. furzeri* [%] | **96.6** | **22.9** | **98.1** | **46.5** | **85.6** |
|  |  |  |  |  |  |  |
| human | length [bp] | 1,871 |  | 157 |  | 5,035 |
|  | identity to *N. furzeri* [%] | **92.8** |  | **95.7** |  | **71.3** |
|  |  |  |  |  |  |  |
|  |  |  |  |  |  |  |

ITS: intergenic transcribed spacer
