# Supplementary material for: High tandem repeat content in the genome of the short-lived annual fish Nothobranchius furzeri: a new vertebrate model for aging research
Source: Genome Biol. 2009 Feb 11;10(2):R16. doi: 10.1186/gb-2009-10-2-r16 (PMC2688266; doi:10.1186/gb-2009-10-2-r16)
Supplement: Additional data file 9 — Genotypes of microsatellites analyzed in N. furzeri strains GRZ and MZM-0403 and the closely related species N. kunthae. [file gb-2009-10-2-r16-S9.doc]

**Additional data file 9: Microsatellite markers in *N. furzeri* and *N. kunthae***

| locus | primers | repeat | alleles [repeat number] | | |
| --- | --- | --- | --- | --- | --- |
|  |  | motif | *N. furzeri* ^1^ | | *N. kunthae* ^2^ |
|  |  |  | GRZ | MZM-0403 |  |
| Nofu0001 | F: GCTACGAGGTTCTGCAGTCA | ATT | 17 | 16, 17,19, 28 | 7, 8, 9 |
|  | R:CACATCCACCTACTAACCAGCA |  |  |  |  |
| Nofu0002 | F: CACCAACTGTGGAGTAGTGC | AC | 33 | 15,20,24,33 | 21, 26, 32, 33 |
|  | R: CCCAACATGAACAAAGACGC |  |  |  |  |
| Nofu0003 | F: TTGGTCACACCTCTGGTTTC | AT | 22 | 20,22,33,38,41 | 28, 30 |
|  | R: CCCAAAGCCAGCTATTAGTCC |  |  |  |  |
| Nofu0004 | F: ACCTATTCCACCTTCCTCAG | TTC | 12 | 10,11,12 | 5, 6, 9 |
|  | R: TCAGAGCTGGGATCTAACC |  |  |  |  |
| Nofu0005 | F: AGTGACCTTGGGTGTTCTG | ATT | 14 | 11,13,14,20 | n.a. |
|  | R: GCTAGGCGGTTAAAGTGTC |  |  |  |  |
| Nofu0006 | F: GGACCACAGAGCAAAAGGAG | AC | 39 | 21,22,27,34,39,44 | 8, 12, 17, 24 |
|  | R: AGTTTTTGCCCCACTGTACG |  |  |  |  |
| Nofu0007 | F: GACTGACTCACAGGGTCAC | AATC | 9 | 5,9 | 5, 7, 15 |
|  | R: CGAATGACCACAGTCACCAA |  |  |  |  |
| Nofu0008 | F: GTTAATCTGGCCCTGCTTGT | GT | 34 | 34,40,41,49,50,53 | n.a. |
|  | R: TGGCACAGGTGAAAGCACTA |  |  |  |  |

^1^ two pools of ten specimens of *N. furzeri* strain GRZ as well as ten specimens of *N. furzeri* strain MZM‑0403 were analyzed; all markers were monomorphic in *N. furzeri* GRZ

^2^ ten specimens of a laboratory strain of *N. kunthae* were analyzed
