# Supplementary material for: High tandem repeat content in the genome of the short-lived annual fish Nothobranchius furzeri: a new vertebrate model for aging research
Source: Genome Biol. 2009 Feb 11;10(2):R16. doi: 10.1186/gb-2009-10-2-r16 (PMC2688266; doi:10.1186/gb-2009-10-2-r16)
Supplement: Additional data file 10 — Analysis of 19 gene-associated markers in N. furzeri strains GRZ and MZM-0403 and N. kunthae. [file gb-2009-10-2-r16-S10.doc]

**Additional data file 10: Gene associated markers in *N. furzeri* and *N. kunthae***

|  |  |  |  |  |  |  |  |
| --- | --- | --- | --- | --- | --- | --- | --- |
| locus | PCR product | gene^2^ | species^2^ | e-value | exonic (E)/ | number of SNPs | |
|  | [bp]^1^ |  |  |  | intronic (I) | MZM-0403 | *N. kunthae* |
|  |  |  |  |  |  |  |  |
| 1 | 573 | *Klf2* | *M. musculus* | 4.0E-030 | E | 1 | 0 |
| 2 | 618 | *AURKB* | *H. sapiens* | 5.9E-051 | I | 1 | 1 |
| 3 | 530 | *Zadh1* | ***P. pygmaeus*** | 6.5E-093 | I | 1 | 1 |
| 4 | 384 | *Sdk2* | *G. gallus* | 1.2E-037 | I | 1 | 0 |
| 5 | 464 | *CSDE1* | *H. sapiens* | 4.4E-058 | E | 1 | 2 |
| 6 | 489 | *GCN1L* | *H. sapiens* | 1.0E-027 | E | 1 | 0 |
| 7 | 965 | *UNC13A* | *H. sapiens* | 3.9E-013 | I | 0 | 4 |
| 8 | 496 | *Tbx1* | *M. musculus* | 3.4E-015 | I | 1 | 0 |
| 9 | 532 | *Sept7* | *R. rattus* | 6.1E-013 | I | 2 | 7 |
| 10 | 484 | *Sufu* | *M. musculus* | 1.7E-015 | I | 3 | 4 |
| 11 | 458 | *MAK* | *H. sapiens* | 6.0E-022 | I | 0 | 1 |
| 12 | 475 | *Sptn2* | *R. rattus* | 1.3E-015 | I | 2 | 0 |
| 13 | 473 | *DLC1* | *H. sapiens* | 1.0E-018 | I | 0 | 7 |
| 14 | 544 | *Zfp-93* | *M. musculus* | 2.0E-015 | I | 1 | 27 |
| 15 | 481 | *ANKRD50* | *H. sapiens* | 1.8E-078 | E | 2 | 2 |
| 16 | 589 | *Retr-1* | *C.elegans* | 5.3E-013 | I | 1 | 4 |
| 17 | 536 | *TMEM1* | *H. sapiens* | 1.1E-030 | I | 2 | 0 |
| 18 | 548 | *FRMD4A* | *H. sapiens* | 7.6E-053 | I | 0 | 7 |
| 19 | 581 | *SODC* | *P. glauca* | 7.3E-016 | I | 0 | 7 |

^1^ size is given for *N. furzeri* GRZ strain which is homozgyous for all analyzed markers

^2^ best BLASTX hit in Swiss-Prot/TrEMBL using the sequence of the *N .furzeri* GRZ strain PCR product as query, gene names are according to name given by the HUGO gene nomenclature commitee (http://www.hugo-international.org/comm_genenomenclaturecommittee.php)
