# Supplementary material for: High tandem repeat content in the genome of the short-lived annual fish Nothobranchius furzeri: a new vertebrate model for aging research
Source: Genome Biol. 2009 Feb 11;10(2):R16. doi: 10.1186/gb-2009-10-2-r16 (PMC2688266; doi:10.1186/gb-2009-10-2-r16)
Supplement: Additional data file 11 — Primers used to clone and sequence five aging related genes in N. furzeri GRZ. [file gb-2009-10-2-r16-S11.doc]

**Additional data file 11: Primer sequences of *N. furzeri* GRZ aging-related genes**

|  | ***Cdkn2b*** |
| --- | --- |
| conserved fw | CAGGTGATGATGATGGG |
| conserved rv | GGCMGCGTCGTGCAGMGG |
| 5Race1 | GGGTGGTCCTGGTGCTCCTGT |
| 5Race2 | TGCTCCTGTCCGCCACGTTC |
| 3Race1 | CAGGTGATGATGATGGGGAGCTG |
| 3Race2 | AGCACGGAGCGGATCCGAACGT |
| Start fw | CCGGATCTCTGTGTGGAC |
| Stop rv | TCGGCTCCTTATTCAGAACC |
|  |  |
|  | ***Cdkn2d*** |
| conserved fw | CTGCAGGTYCTGGTGGAG |
| conserved rv | GCCTTCYCGKATGGCGTA |
| 5Race1 | GGCGCCAGGAACTTCACCAC |
| 5Race2 | CTTCTCGGATGGCGATGTGAATG |
| 3Race1 | AGCACGGGGCTTCGGTGAAC |
| 3Race2 | GAACCTTCCCGACCACAATGGAG |
| Start fw | GCAGGAACAGAGACTATCATGG |
| Stop rv | CAATCTTATGAACTGTGGACGTG |
|  |  |
|  | ***Msra*** |
| conserved fw | GCAGAGAGRAARYTCTGGAG |
| conserved rv | AGDCCRCAGTASCCATCRG |
| 5Race1 | GGTACTGTTGGTGGTAGTCCTCAGCA |
| 5Race2 | AGTAATTGGGCCAAACCCGTCCT |
| 3Race1 | CACCCAGGTGGGCTACTGTGGAG |
| 3Race2 | AAGGAGGTGTGCACAGGCAGGAC |
| Start fw | ATTGGAGACGGACTTTGGTG |
| Stop rv | TTCATTTCTCTTGTTGCTCTGC |
|  |  |
|  | ***Sirt1*** |
| conserved fw | based on Genade et al. 2005 |
| conserved rv | based on Genade et al. 2005 |
| 5Race1 | GTCAGGTTTCATGATTGCCAGAGG |
| 5Race2 | CATCTTCCCTTATAGCTTCAC |
| 3Race1 | TCAGACGGGACCCTAGACCCTTT |
| 3Race2 | AGTGGCTGGAGTTCAGAGGA |
| Start fw | GTTGAAGATGGCGGATGAAG |
| Stop rv | GGAGTCTTATTACGCTTGATTCG |
|  |  |
|  | ***Tp53*** |
| conserved fw | CACCACCARAACGAGGACT |
| conserved rv | GTTCATSCCCCCCATGCA |
| 5Race1 | ggtcgtcatttctgagcccaactg |
| 5Race2 | gggggctcataaggcacagtcac |
| 3Race1 | aagtcatttgatccgcgttgagg |
| 3Race2 | ccagctggctcagtactttgagga |
| Start fw | TCGCTAAAACGAAAGCAAGTC |
| Stop rv | ACGGCCTCAGAGAACAAAAC |
